# hapbin: An Efficient Program for Performing Haplotype-Based Scans for Positive Selection in Large Genomic Datasets

**DOI:** 10.1093/molbev/msv172

**Published:** 2015-08-06

**Authors:** Colin A. Maclean, Neil P. Chue Hong, James G.D. Prendergast

**Affiliations:** ^1^EPCC, School of Physics and Astronomy, University of Edinburgh, Edinburgh, Scotland, United Kingdom; ^2^Roslin Institute, University of Edinburgh, Scotland, United Kingdom

**Keywords:** selection, iHS, EHH, XP-EHH, software

## Abstract

Understanding how the genome is shaped by selective processes forms an integral part of modern biology. However, as genomic datasets continue to grow larger it is becoming increasingly difficult to apply traditional statistics for detecting signatures of selection to these cohorts. There is therefore a pressing need for the development of the next generation of computational and analytical tools for detecting signatures of selection in large genomic datasets. Here, we present hapbin, an efficient multithreaded implementation of extended haplotype homzygosity-based statistics for detecting selection, which is up to 3,400 times faster than the current fastest implementations of these algorithms.

As a selected allele is swept through a population the haplotype on which it resides will increase in frequency faster than recombination can break it down. As a result alleles under positive selection will be expected to reside upon unusually long haplotypes given their frequency, and such extended haplotype homozygosity (EHH) (Sabeti et al. 2002) forms the basis of a number of the most popular tests of selection including the integrated haplotype score (iHS) (Voight et al. 2006) and the cross-population EHH (XP-EHH) statistic (Sabeti et al. 2007). These haplotype-based methods of detecting selection have a number of advantages over other tests; for example their ability to detect partial or incomplete sweeps (Vitti et al. 2013), short-term balancing selection (Vitti et al. 2013) and their comparative insensitivity to background selection (reduced neutral variation as a result of purifying selection at linked deleterious sites) that can otherwise confound studies of adaptive evolution (Enard et al. 2014). However with sequencing costs falling faster than computational speeds are increasing (Check Hayden 2014), as genomic datasets grow larger it is becoming increasingly difficult to apply these statistics to contemporary cohorts.

Recently an improved implementation of these statistics was made available within the selscan program (Szpiech and Hernandez 2014), demonstrated to be two times faster at calculating iHS than the next fastest algorithm, rehh (Gautier and Vitalis 2012). However even with this improved implementation of these statistics the calculation of iHS across 100 whole human genomes, the approximate average size of a 1000 genomes (1000 Genomes Project Consortium et al. 2010) population cohort, is still expected to take over 2 months when run on a single core on a standard desktop machine. For these algorithms to be widely used, there is a requirement for the development of new, faster, and more efficient, computational approaches to improve the speed at which EHH-based selection scans can be carried out. As a result, allowing for the analysis of whole-genome sequencing datasets of ever increasing size to be processed in reasonable timeframes and on non-specialist hardware.

Here, we introduce hapbin that utilizes a new computational approach (see Supplementary methods, Supplementary Material online) to calculate the EHH, iHS, and XP-EHH statistics. We show that this implementation is up to 3,400 times faster than even selscan, allowing iHS to be calculated across 100 fully sequenced human genomes in ∼3 h, as opposed to over 2 months, when run on a single core on a standard desktop machine.

To assess the performance of hapbin, it was first benchmarked alongside selscan on two different hardware architectures. An Amazon c3.8xlarge EC2 Ubuntu instance (32 CPUs) as well as on ARCHER; the UK National Supercomputer. Importantly hapbin will equally run on a standard desktop machine but the use of these resources allowed us to assess its scalability while also enabling other users to repeat these analyses. Performance was characterized using various subsets of data from chromosome 22 of the 1000 genomes project (1000 Genomes Project Consortium et al. 2010) cohort (phase 1 version 3) and both programs were run with default parameters (an EHH decay cutoff of 0.05 and minimum minor allele frequency of 5%).

As shown in figures 1*A*–*C*, Supplementary figure S1 and table S1, Supplementary Material online, hapbin is from 88 to 3,400 times quicker than selscan at calculating the iHS, depending on the hardware used and the number of variants and individuals in the input dataset. With an input cohort of 50 individuals hapbin processed all 489,301 genetic variants on chromosome 22 in 37 s when run across one core on ARCHER. In comparison, selscan took 35 h. As shown in figure 1*D,* this speedup comes with no loss of accuracy.

**Fig. 1.**
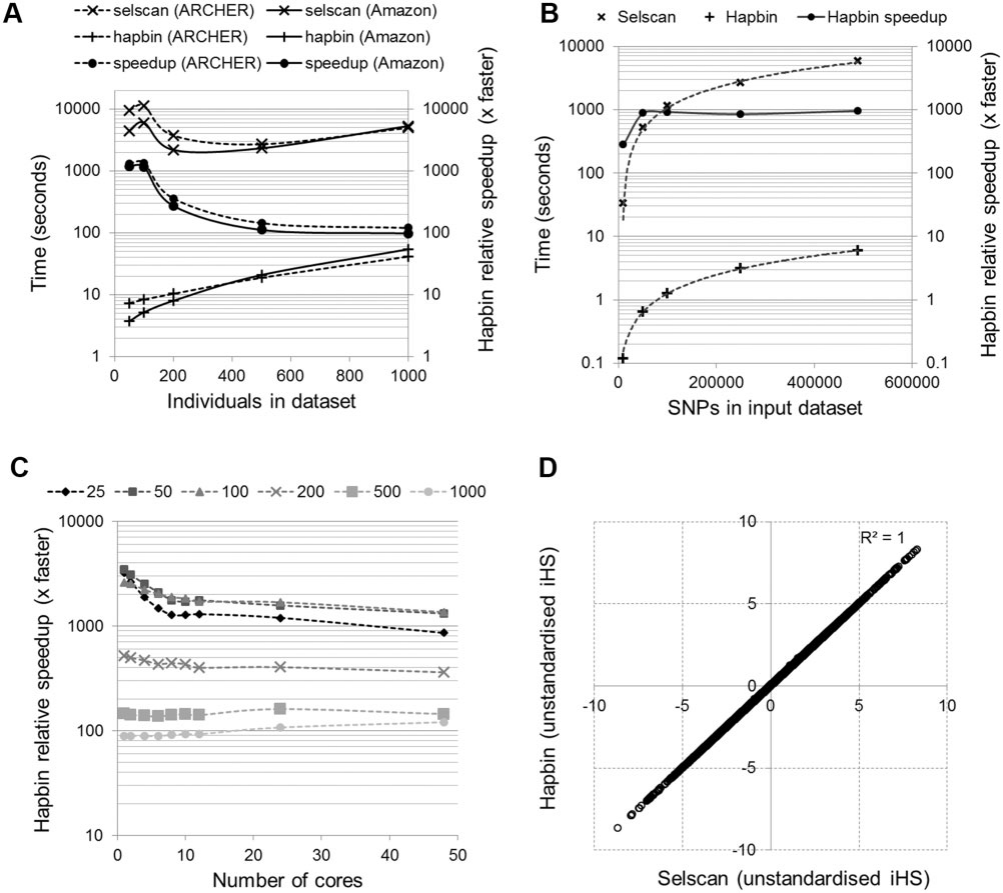
Hapbin versus selscan comparisons. (*A*) Time taken by hapbin and selscan to calculate iHS across chromosome 22 across 48 cores (1 node) onz ARCHER and on an Amazon c3.8xlarge instance. Subsets of individuals being randomly sampled from the 1000 genomes dataset. (*B*) Time taken by hapbin and selscan to calculate iHS in the 1000 genomes GBR (Great Britain) population of 89 individuals on the Amazon c3.8xlarge instance. Runs of contiguous SNPs by location were subsampled from all of those on chromosome 22. (*C*) Hapbin’s relative speedup versus selscan when run across chromosome 22 with varying numbers of cores and individuals on ARCHER. (*D*) Comparison of unstandardized iHS values output by both selscan and hapbin when run across 500 randomly selected individuals and all SNPs on chromosome 22.

A further advantage of hapbin is that while selscan requires a further program to produce standardized iHS from the unstandardized values it outputs, hapbin produces both by default. As a result all selscan timings presented here are the times taken to calculate unstandardized iHS only, while those for hapbin are for the calculation of both standardized and unstandardized values. Hapbin’s relative speedup at calculating XP-EHH with respect to selscan are more modest than those observed when calculating iHS but order of magnitude speedups are still observed (Supplementary fig. S1, Supplementary Material online).

The hapbin program can be downloaded from https://github.com/evotools/hapbin (last accessed August 10, 2015). Hapbin can be applied to datasets from any meiotically recombinant species and takes phased genotypes in IMPUTE format (Howie et al. 2009), as produced by the popular phasing algorithm SHAPEIT2 (O’Connell et al. 2014). To accompany the program, we have also exploited the speed of hapbin to calculate iHS genome-wide for all 26 populations in the recently released, phased, 1000 genomes phase 3 cohort (Delaneau et al. 2014). These can be downloaded from http://dx.doi.org/10.7488/ds/214 (last accessed August 10, 2015) or viewed at the UCSC genome browser (Supplementary figs. S2 and S3, Supplementary Material online).

## Supplementary Material

Supplementary methods, figures S1–S3, and table S1 are available at *Molecular Biology and Evolution* online (http://www.mbe.oxfordjournals.org/).

Supplementary Data
