# Supplementary material for: hapbin: An Efficient Program for Performing Haplotype-Based Scans for Positive Selection in Large Genomic Datasets
Source: Mol Biol Evol. 2015 Aug 6;32(11):3027–9. doi: 10.1093/molbev/msv172 (PMC4651233; doi:10.1093/molbev/msv172)
Supplement: Supplementary Data [file supp_32_11_3027__index.html]

hapbin: An Efficient Program for Performing Haplotype-Based Scans for Positive Selection in Large Genomic Datasets — hapbin: An Efficient Program for Performing Haplotype-Based Scans for Positive Selection in Large Genomic Datasets — Supplementary Data 

# hapbin: An Efficient Program for Performing Haplotype-Based Scans for Positive Selection in Large Genomic Datasets

## Supplementary Data

files

- Supplementary Data - pdf file
