# Supplementary material for: hapbin: An Efficient Program for Performing Haplotype-Based Scans for Positive Selection in Large Genomic Datasets
Source: Mol Biol Evol. 2015 Aug 6;32(11):3027–9. doi: 10.1093/molbev/msv172 (PMC4651233; doi:10.1093/molbev/msv172)
Supplement: Supplementary Data [file supp_msv172_SuppMBE_final.pdf]

hapbin: An efficient program for performing haplotype based scans for positive selection in large genomic datasets.

Supplementary Methods, Table 1 and Figures 1-3

Supplementary Methods

At their core, all EHH based tests involve examining how haplotype identity decays across a group of individuals with increasing distance from an allele of interest. Following the phasing of haplotypes, one of the major rate limiting factors of implementations of these statistics is the inefficiency with which they store and access the genetic data to characterise this haplotype decay. Encoding the variants of a whole genome genetic dataset of ~1000 individuals as a space or tab separated text file requires greater than 160Gb, whereas encoding it as a binary file reduces this by up to 16 fold (the allele and space representation of 2x8 bits being reduced to 1 bit). Once stored as a binary representation it is then possible to improve the efficiency of the EHH algorithm through the use of bitwise operations to process many alleles for each CPU instruction. Determining EHH is based upon identifying the frequency of each haplotype combination of a given length, n. The smaller the number of observed haplotypes at length n, in general, the higher the EHH. Rather than explicitly traversing along each haplotype and storing each subhaplotype of length n along with a corresponding count, hapbin instead represents the *divergence* of haplotypes in the form of a binary haplotype tree. The frequencies of each haplotype of a given length can then be determined, as described in greater detail below, without having to explicitly store the haplotypes themselves. By storing the genetic data as a binary file and determining haplotype frequencies through the use of a bitwise algorithm, a reduced memory footprint and substantially faster runtime is achieved in comparison to other implementations.

In greater detail, in the IMPUTE hap file format (https://mathgen.stats.ox.ac.uk/impute/impute_v2.html#-h) alleles at biallelic variants are encoded as either '0' or '1', with each variant separated by a newline character and each allele separated by a space. Each line in a hap formatted file consequently representing a variant and each column representing a phased chromosome haplotype. An immediate observation of the .hap data format is the binary nature of the data. The spaces between alleles are also unnecessary, causing the .hap files to require roughly twice as much memory as actually required. Each '0' and '1' character also takes up a full byte but can instead be stored as bits. By representing SNPs as a set of bits rather than characters, the storage requirement can be reduced by roughly 16x: 2 characters at 8 bits per character reduced to 1 bit.

There is, however, a significant challenge to storing SNPs as a set of bits: the algorithm to calculate EHH reads along both the rows and columns of the data. Furthermore, memory is addressed in byte sized chunks, not bits. In order to identify if a particular allele is 0 or 1, the relevant bit must be extracted. This can be done using a bitmask. A bitmask is set of bits used to select or toggle a certain bit or set of bits from another set of bits. A bitmask can be used to select desired bits using the bitwise AND operator.

The key to an efficient bitwise haplotype identification algorithm is using a bitset to store a mask of the locations of the haplotypes, not the haplotypes themselves. For each haplotype, h, observed, let $B_{N,h}$ be a bitset of length N indicating the position of haplotype h. The indices of the 1 bits in $B_{N,h}$ correspond to the zero-indexed chromosomes where the haplotype is observed. For example if a bitset is denoted by $[b_0b_1b_2...b_{N-1}]_N$ where b_n is the bit at position n and N is the number of bits in the bitset, then the haplotype "000" in Supplementary Methods Figure 1 has the corresponding bitset $B_{8,000} = [11001000]_8$ i.e. the 000 haplotype is observed in the first, second and fifth columns (Note: the binary digits are read from right to left but the data is read from left to right.)

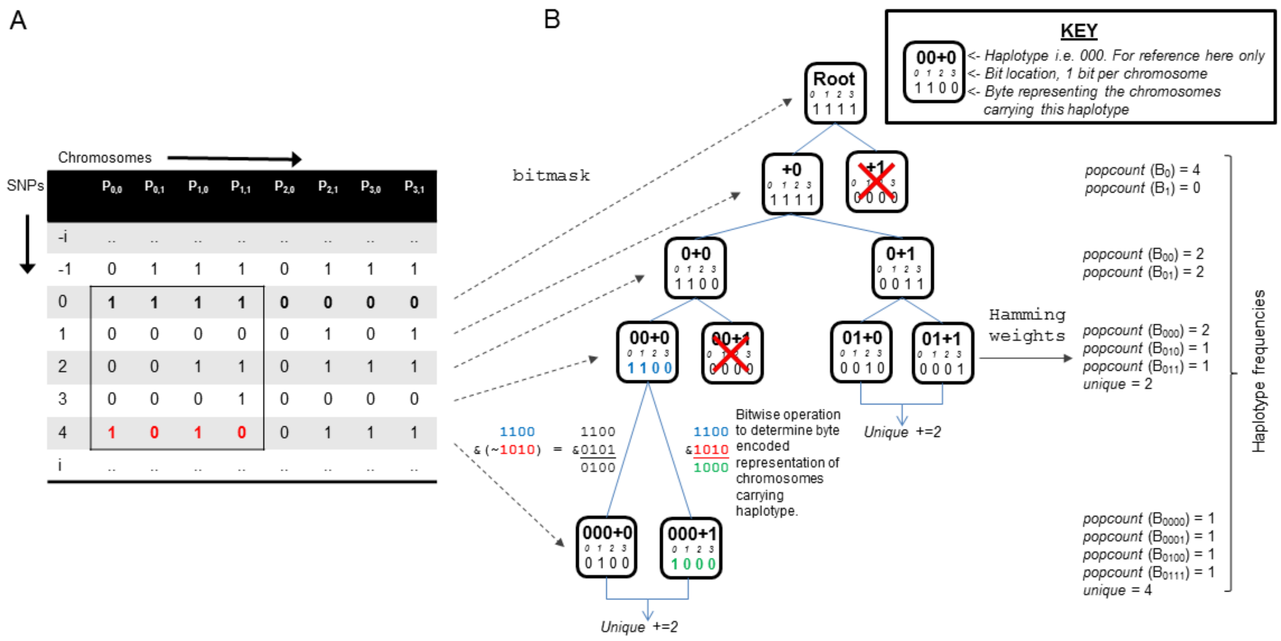

Supplementary Methods Figure 1: (A) Binary encoding of diploid genetic data. Four individuals and 6 SNPs are shown with $P_{0,0}$ and $P_{0,1}$ corresponding to the two chromosome of individual 0. Example SNP of interest around which EHH is to be calculated is located at position 0 with alleles in bold. (B) Bitwise haplotype tree where the "1" allele is present at the SNP of interest (corresponding to boxed alleles in (A)). To determine the frequency of the haplotypes of length n in the population downstream from the SNP of interest, bitmasks are used to isolate binary representation of the alleles present at SNP n. These are then combined with the previously calculated frequencies observed of haplotypes of length n-1 through the use of bitwise operations. For example B_{000}=1100 (coloured in blue in (B)), indicating that of the four chromosomes, the first two carry the 000 haplotype between SNPs 1 and 3. The individuals that carry the 0001 haplotype will therefore be 1100 & 1010 = 1000 where & corresponds to the bitwise AND (1010 being the allelic representation of SNP 4, coloured in red in (A)). Therefore B_{0001}=1000 (green in (B)), indicating only the first chromosome carries the 0001 haplotype. The frequency of this haplotype is retrieved by determining the Hamming weight of this returned byte, which in this case is 1.

A haplotype, h, of length i can become h + 0 or h + 1 at length i + 1. Using bitwise operations, these possible divergences can be processed for an entire bitset at once:

$$B_{N,h+0} = B_{N,h} \ \& \sim B_{N,x_{i+1}}$$
$$B_{N,h+1} = B_{N,h} \ \& \ B_{N,x_{i+1}}$$

Equations 1,2

where $B_{N,xi+1}$ is a bitset containing the data at marker x_{i+1}. For example, using the data from Supplementary Methods Figure 1:

$$
\begin{aligned}
B_{8,x_4} &= [10100111]_8 \\
B_{8,0000} &= B_{8,000} \mathbin{\&} (\sim B_{8,x_4}) \\
&= [11001000]_8 \mathbin{\&} (\sim [10100111]_8) \\
&= [11001000]_8 \mathbin{\&} [01011000]_8 \\
&= [01001000]_8 \\
B_{8,0001} &= B_{8,000} \mathbin{\&} B_{8,x_4} \\
&= [11001000]_8 \mathbin{\&} [10100111]_8 \\
&= [10000000]_8
\end{aligned}
$$

Equations 3,4,5

An algorithm, popcount is then used to count the occurrences of a given haplotype. This operation counts the number of 1 bits set in an integer, also known as the Hamming weight. For example popcount($[11010011]_8$) = 5, i.e. 5 chromosomes carry this haplotype. There are algorithms which can perform the popcount operation in fewer steps than individually testing if each bit is set to 1. Furthermore, modern x86 CPUs have a POPCNT instruction which can perform this operation in a single clock cycle. If popcount ($B_{N,h}$) = 0, then the haplotype, h, is not observed in the data. All of the observed haplotypes at marker x_i can be stored as a set of bitsets R_i and all of the haplotypes at marker x_{i+1}, R_{i+1}, can be found by applying equations 1 and 2 to each element of R_i. The set of bitsets at index i = 0 shall be defined as containing a bitset with all bits set: $R_0 = \{[11...1]_N\}$.

Thus, a recursive bitwise haplotype identification algorithm can be defined as:

$$
\mathcal{R}_0 = \{[11\cdots 1]_N\}
$$

$$
\mathcal{R}_i = \begin{cases} \{b \mathbin{\&} (\sim B_{N,x_i})\,|b \in \mathcal{R}_{i-1}\} \cup \{b \mathbin{\&} B_{N,x_i}|b \in \mathcal{R}_{i-1}\} & |i > 0 \\ \{b \mathbin{\&} (\sim B_{N,x_i})\,|b \in \mathcal{R}_{i+1}\} \cup \{b \mathbin{\&} B_{N,x_i}|b \in \mathcal{R}_{i+1}\} & |i < 0 \end{cases}
$$

This algorithm can be optimized by excluding bitsets with a population count of 0 since subsequent iterations will also always have a population count of 0. Since unique haplotypes can diverge no further, bitsets with a population count of 1 can be removed from the tree, counted, and factored in separately. It is also unnecessary to be able to reconstruct what the specific sequence of the haplotypes are. All that is necessary is knowing the locations of the different haplotypes. Therefore, previous levels of the tree can be discarded once the level's EHH and next level are calculated and there is no need to store relational information between nodes, saving memory.

From the tree of haplotype locations, it is straightforward to calculate the EHH for a particular length. Because the locations at which a haplotype occurs are represented by '1' bits at positions corresponding to the chromosomes, the number of times a haplotype is observed can be computed by finding the population count of the bitset. The EHH for core haplotype c can then be found as previously described (Sabeti et al. 2002):

$$
EHH_c(x_i) = \sum_{h \in \mathcal{H}_c(x_i)} \left(\frac{n_h}{n_c}\right)^2
$$

where h is each haplotype in the set $H_c(x_i)$,n_h is the number of times h is observed, and n_c is the number of times the core haplotype, c, is observed at the location of interest. The n_h for haplotype h in combination with core haplotype c can therefore be found by n_h = popcount ($B_{N,h}$ & $B_{N,c}$). The core haplotypes are added at the end in order to reduce the complexity of the tree. iHS and XP-EHH can then be calculated from these EHH values as previously described (Voight et al. 2006; Sabeti et al. 2007).

Supplementary Table

Number of haplotypes (i.e. 2 x number of individuals)	Cores	Selscan runtime	Hapbin runtime
50	1	20h51m4.531s	0h0m23.225s
50	2	10h48m53.646s	0h0m14.775s
50	4	5h46m57.750s	0h0m11.118s
50	6	4h7m58.826s	0h0m10.076s
50	8	3h9m56.171s	0h0m8.976s
50	10	2h40m44.689s	0h0m7.612s
50	12	2h42m4.777s	0h0m7.534s
50	24	2h12m52.744s	0h0m6.691s
50	48	1h50m3.065s	0h0m7.715s
100	1	35h47m1.343s	0h0m37.103s
100	2	18h32m49.265s	0h0m21.756s
100	4	9h54m39.405s	0h0m14.218s
100	6	7h2m40.502s	0h0m12.165s
100	8	5h24m9.480s	0h0m11.049s
100	10	4h45m14.126s	0h0m10.097s
100	12	4h18m47.401s	0h0m8.869s
100	24	3h17m32.393s	0h0m7.577s
100	48	2h40m18.798s	0h0m7.337s
200	1	48h00m00s	0h1m6.477s
200	2	25h48m53.135s	0h0m36.790s
200	4	13h48m10.871s	0h0m22.524s
200	6	9h49m6.705s	0h0m17.550s
200	8	7h31m44.579s	0h0m14.407s
200	10	6h34m4.275s	0h0m13.002s
200	12	5h36m16.819s	0h0m11.874s
200	24	3h58m45.380s	0h0m8.545s
200	48	3h8m51.046s	0h0m8.378s
400	1	16h45m56.222s	0h1m56.229s
400	2	8h38m57.750s	0h1m2.914s
400	4	4h39m57.174s	0h0m35.989s
400	6	3h19m23.787s	0h0m27.736s
400	8	2h40m31.673s	0h0m21.847s
400	10	2h13m56.369s	0h0m18.720s
400	12	1h53m35.200s	0h0m17.126s
400	24	1h15m45.106s	0h0m11.289s
400	48	1h2m16.674s	0h0m10.390s
500	1	8h27m44.160s	0h2m7.529s
500	2	4h22m54.812s	0h1m8.533s
500	4	2h20m18.069s	0h0m39.605s
500	6	1h40m4.625s	0h0m30.144s
500	8	1h19m42.881s	0h0m23.580s

500	10	1h7m37.705s	0h0m20.744s
500	12	0h57m25.981s	0h0m17.885s
500	24	0h39m53.598s	0h0m11.886s
500	48	0h30m50.915s	0h0m10.576s
1000	1	13h34m51.695s	0h5m39.481s
1000	2	7h1m11.983s	0h2m59.102s
1000	4	3h44m58.369s	0h1m37.702s
1000	6	2h41m13.809s	0h1m10.990s
1000	8	2h7m19.301s	0h0m54.220s
1000	10	1h46m22.113s	0h0m44.814s
1000	12	1h29m15.726s	0h0m38.164s
1000	24	0h59m13.104s	0h0m22.154s
1000	48	0h45m7.001s	0h0m18.829s
2000	1	24h20m40.874s	0h16m23.778s
2000	2	12h37m42.237s	0h8m29.152s
2000	4	6h44m55.451s	0h4m33.949s
2000	6	4h52m20.568s	0h3m18.110s
2000	8	3h45m40.006s	0h2m28.769s
2000	10	3h6m17.850s	0h2m0.472s
2000	12	2h36m9.052s	0h1m41.501s
2000	24	1h36m52.302s	0h0m54.355s
2000	48	1h23m6.782s	0h0m41.470s

Supplementary Table 1: Time taken by selscan and hapbin to calculate iHS across chromosome 22 on ARCHER with various subsets of the 1000 genomes cohort and when run across various numbers of threads.

Supplementary Figures

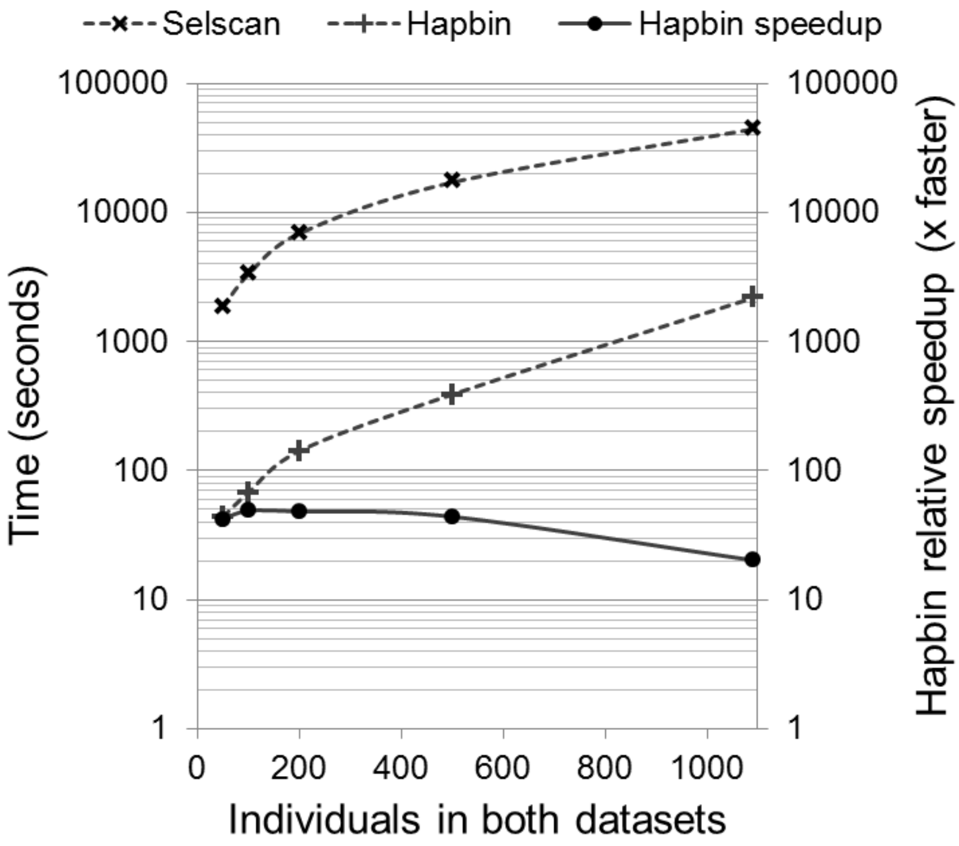

Supplementary Figure 1: Time taken by selscan and hapbin to calculate XP-EHH across chromosome 22 with various subsets of the 1000 genomes cohort (Amazon c3.8xlarge instance).

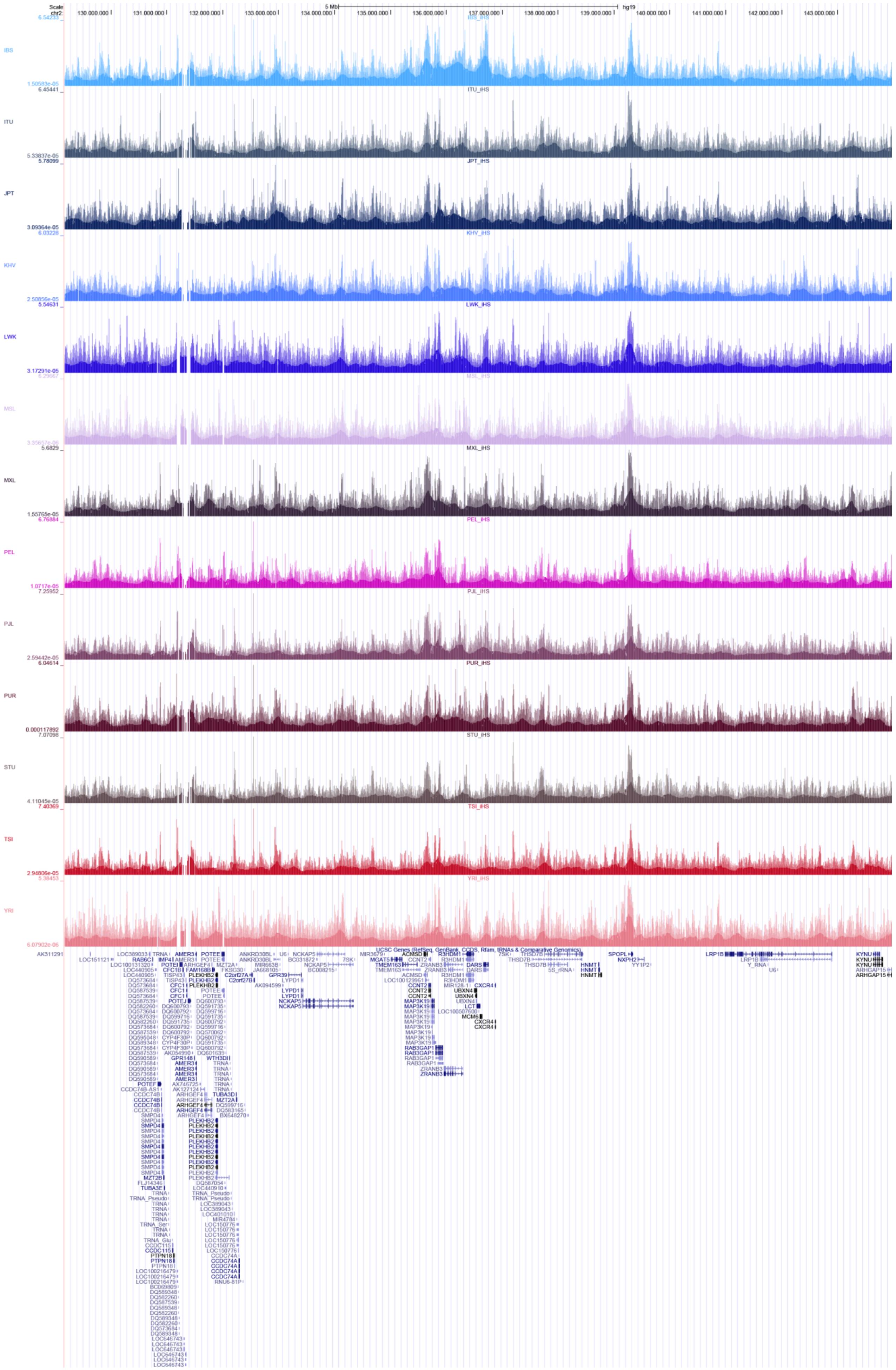

Scale
chr2:
5 Mb
hg19
130,000,000
131,000,000
132,000,000
133,000,000
134,000,000
135,000,000
136,000,000
137,000,000
138,000,000
139,000,000
140,000,000
141,000,000
142,000,000
143,000,000
IBS
IBS_iHS
ITU
ITU_iHS
JPT
JPT_iHS
KHV
KHV_iHS
LWK
LWK_iHS
MSL
MSL_iHS
MXL
MXL_iHS
PEL
PEL_iHS
PJL
PJL_iHS
PUR
PUR_iHS
STU
STU_iHS
TSI
TSI_iHS
YRI
YRI_iHS
UCSC Genes (RefSeq, GenBank, CCDS, Rfam, tRNAs & Comparative Genomics)

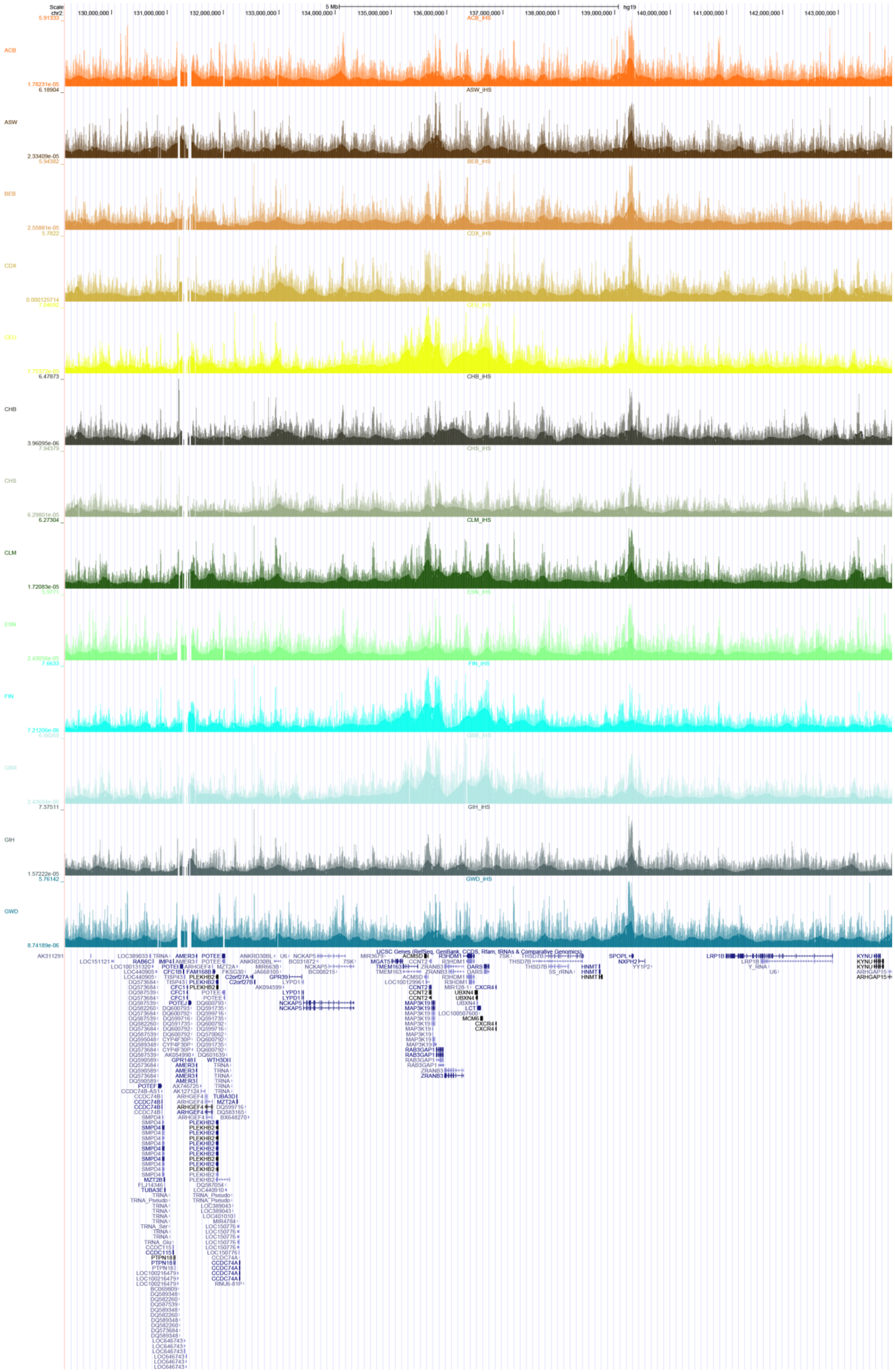

Scale
chr2:
5 Mb
hg19
130,000,000
131,000,000
132,000,000
133,000,000
134,000,000
135,000,000
136,000,000
137,000,000
138,000,000
139,000,000
140,000,000
141,000,000
142,000,000
143,000,000
ACB
ACB_iHS
5.91333
1.78231e-05
ASW
ASW_iHS
6.18904
2.33409e-05
BEB
BEB_iHS
5.94382
2.55881e-05
CDX
CDX_iHS
5.7822
0.000125714
CEU
CHB
CHB_iHS
6.47873
3.96095e-06
CHS
CHS_iHS
7.94379
6.29801e-05
CLM
CLM_iHS
6.27304
1.72083e-05
ESN
ESN_iHS
FIN
FIN_iHS
7.6633
7.21206e-06
GBR
GIH
GIH_iHS
7.37511
1.57222e-05
GWD
GWD_iHS
5.76142
8.74189e-06
UCSC Genes (RefSeq, GenBank, CCDS, Rfam, tRNAs & Comparative Genomics)

Supplementary Figures 2 and 3: Happbin iHS results for all 26 populations in the phase 3 version of the 1000 genomes project. The region around the lactase (LCT) gene on chromosome 2 is shown. Elevated iHS in and around this gene can be seen in the CEU, IBS, FIN and GBR European populations, in agreement with the known recent positive selection at this locus in European populations. In agreement with previous studies little evidence of selection was observed at this locus in the final European population from Tuscany (TSI) (Schlebusch et al. 2013). These genome-wide iHS can be downloaded from http://dx.doi.org/10.7488/ds/214. Alternatively the files can be viewed directly at UCSC by pasting the URL of the files (e.g. http://datashare.is.ed.ac.uk/bitstream/handle/10283/714/ASW_chr1.bg.gz) into the corresponding box here: http://genome-euro.ucsc.edu/cgi-bin/hgCustom?clade=mammal&org=Human&db=hg37.

References

Sabeti PC, Reich DE, Higgins JM, Levine HZP, Richter DJ, Schaffner SF, Gabriel SB, Platko JV, Patterson NJ, McDonald GJ, et al. 2002. Detecting recent positive selection in the human genome from haplotype structure. Nature. 419:832–837.

Sabeti PC, Varilly P, Fry B, Lohmueller J, Hostetter E, Cotsapas C, Xie X, Byrne EH, McCarroll SA, Gaudet R, et al. 2007. Genome-wide detection and characterization of positive selection in human populations. Nature. 449:913–918.

Schlebusch CM, Sjödin P, Skoglund P, Jakobsson M. 2013. Stronger signal of recent selection for lactase persistence in Maasai than in Europeans. Eur J Hum Genet. 21:550–553.

Voight BF, Kudaravalli S, Wen X, Pritchard JK. 2006. A map of recent positive selection in the human genome. PLoS Biol. 4:e72.
